# Impact of high Fe-concentrations on microbial community structure and dissolved organics in hydrothermal plumes: an experimental study

**DOI:** 10.1038/s41598-022-25320-0

**Published:** 2022-12-01

**Authors:** Christian T. Hansen, Charlotte Kleint, Stefanie Böhnke, Lukas Klose, Nicole Adam-Beyer, Katharina Sass, Rebecca Zitoun, Sylvia G. Sander, Daniela Indenbirken, Thorsten Dittmar, Andrea Koschinsky, Mirjam Perner

**Affiliations:** 1 Institute for Chemistry and Biology of the Marine Environment (ICBM), Carl Von Ossietzky University of Oldenburg, Oldenburg, Germany; 2 Center for Marine Environmental Sciences (MARUM), University of Bremen, Bremen, Germany; 3 Department of Physics & Earth Sciences, Jacobs University Bremen, Bremen, Germany; 4 Geomicrobiology, Department of Marine Biogeochemistry, GEOMAR Helmholtz Centre for Ocean Research, Kiel, Germany; 5 Molecular Biology of Microbial Consortia, Biocenter Klein Flottbek, University of Hamburg, Hamburg, Germany; 6 Heinrich-Pette-Institut, Leibniz Institute for Experimental Virology, Martinistraße 52, 20251 Hamburg, Germany; 7 Department of Chemistry, University of Otago, Dunedin, 9054 New Zealand; 8 Marine Mineral Resource Group, GEOMAR Helmholtz Centre for Ocean Research Kiel, 24148 Kiel, Germany

**Keywords:** Biogeochemistry, Environmental chemistry, Marine chemistry, Microbiology

## Abstract

Iron (Fe) is an essential trace element for life. In the ocean, Fe can be exceptionally scarce and thus biolimiting or extremely enriched causing microbial stress. The ability of hydrothermal plume microbes to counteract unfavorable Fe-concentrations up to 10 mM is investigated through experiments. While Campylobacterota (*Sulfurimonas*) are prominent in a diverse community at low to intermediate Fe-concentrations, the highest 10 mM Fe-level is phylogenetically less diverse and dominated by the SUP05 clade (Gammaproteobacteria)*,* a species known to be genetically well equipped to strive in high-Fe environments. In all incubations, Fe-binding ligands were produced in excess of the corresponding Fe-concentration level, possibly facilitating biological Fe-uptake in low-Fe incubations and detoxification in high-Fe incubations. The diversity of Fe-containing formulae among dissolved organics (SPE-DOM) decreased with increasing Fe-concentration, which may reflect toxic conditions of the high-Fe treatments. A DOM-derived degradation index (I_DEG_) points to a degradation magnitude (microbial activity) that decreases with Fe and/or selective Fe-DOM coagulation. Our results show that some hydrothermal microbes (especially Gammaproteobacteria) have the capacity to thrive even at unfavorably high Fe-concentrations. These ligand-producing microbes could hence play a key role in keeping Fe in solution, particularly in environments, where Fe precipitation dominates and toxic conditions prevail.

## Introduction

Iron (Fe) is a fundamental trace nutrient regulating phytoplankton productivity in the upper water column and thus affecting the biological carbon pump^1^. Although Fe is considerably scarce in most parts of the ocean, in certain habitats (e.g. hydrothermal vents) its concentrations can exceed levels that stress microbial life, where minerals precipitate in the cell’s periplasm or on the cell surface resulting in irreversible encrustation and cell death^2,3^. Some microbes have evolved mechanisms to bind Fe, e.g., via active production of organic molecules, so called ligands, to condition the environment in their favor. Organic ligands include e.g., siderophores, polyphenols, hemophores and heme and can either enhance Fe-bioavailability or be used as detoxification tools via complexation^4–7^. Fe-complexation with organic ligands also effectively prevents rapid precipitation in seawater. The thereby resulting increased residence time enables Fe-export over greater distances and thus enhances Fe-transfer to the deep sea or the surface ocean^8–10^. In addition, the systematics of coupled Fe-oxidation and Fe-organic complexation can influence toxicity of other metals and metalloids (e.g. sorption of cadmium or arsenic to Fe-precipitates) as well as the accumulation of toxic nitrite (if Fe(II) is not complexed)^11^.

**Figure 1:**
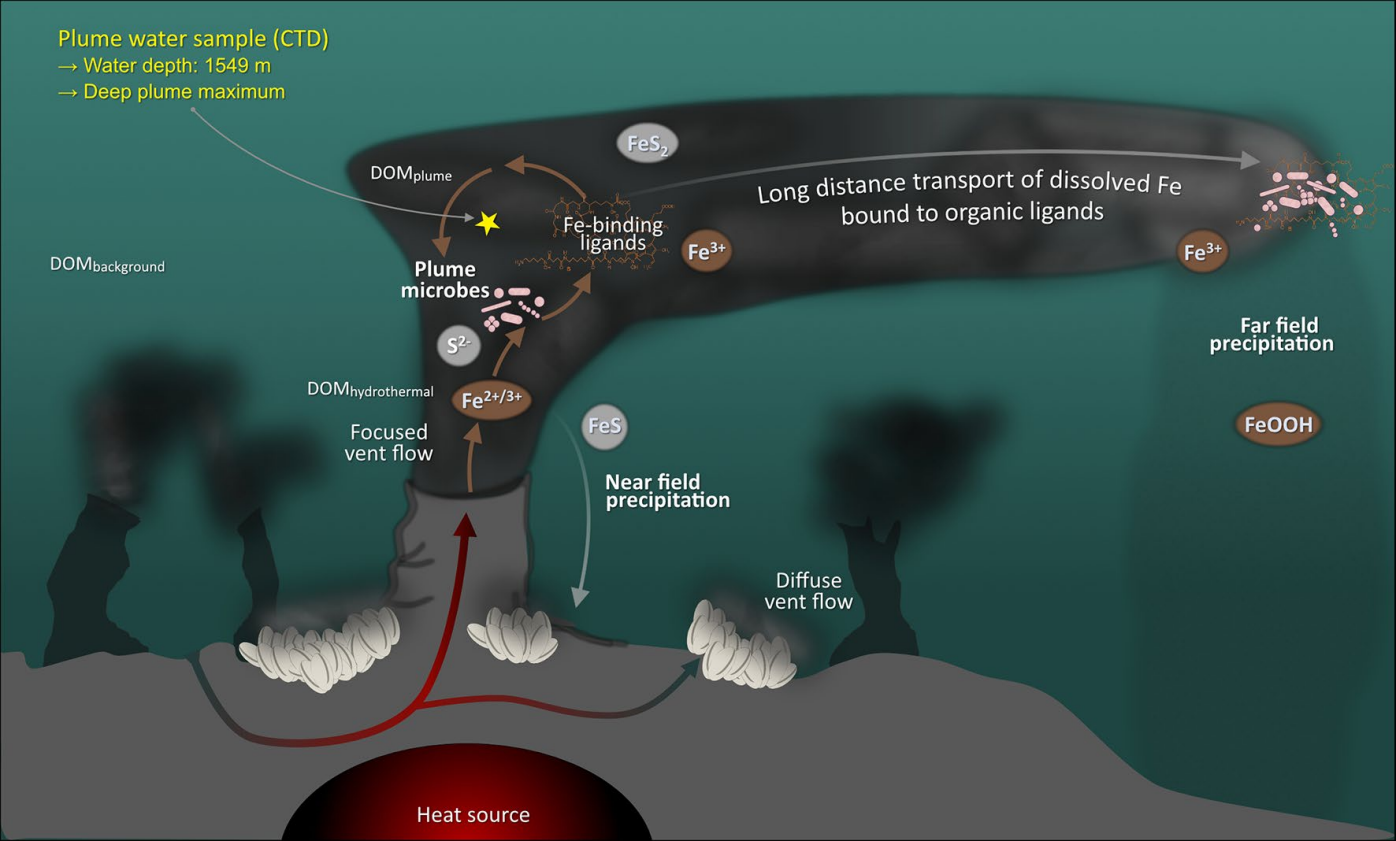
Schematic sketch of the importance of Fe within a plume environment including parameters considered in this study. Shown is a hot, focused Fe-enriched hydrothermal fluid vent with associated buoyant plume. Within the vent and plume, endemic microbes interact with dissolved organics (DOM) and potentially produce Fe-binding ligands. These interactions are crucial for the potential of Fe being exported away from the vent source. The bulk plume water sample (yellow star) used to set up the incubated dilutions (50 mL plume sample + 450 mL artificial seawater medium + individual Fe amendment) was collected using a rosette water sampler equipped with 20 L bottles and a CTD sensor from the deep plume maximum at 1549 m water depth at Brothers volcano.

Besides dust, rivers, and sediments, hydrothermal vents are one of the dominant sources of Fe to the ocean (see Fig. 1)^9,12–15^. For a long time, it was assumed that Fe precipitates nearby vent outlets. However, in recent years, several studies have shown that organically bound Fe can be stabilized and transported over long distances of up to 4000 km in the open ocean^10,16^. Hydrothermal vents are also sources of dissolved organic matter (DOM)^17,18^, which potentially include Fe-binding ligands^19,20^, and host specialized microbial communities^21^ that might be able to reduce Fe-toxicity by actively producing Fe-binding organic ligands. An analogous ability has been demonstrated by vent microbes of the Mid-Atlantic Ridge in laboratory-based experiments with copper (Cu)^22^ at levels above the required threshold, and similar processes could be assumed for Fe. This assumption is supported by field studies quantifying Fe-binding ligands in hydrothermal plumes and geochemical models, both confirming that organic ligands are crucial for the distribution of hydrothermal Fe in the water column and for mediating Fe-availability to marine microbes^8,23,24^.

**Figure 2:**
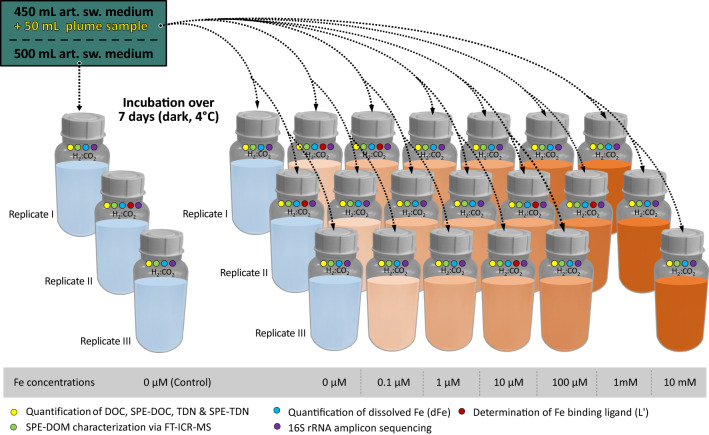
Design of the conducted 7-day incubation experiments. Triplicates (one replicate was lost for 1 mM Fe-level) consisting of 50 mL plume sample plus 450 mL artificial seawater medium (ASW) were set up for a range of spiked Fe-concentrations (Fe(II)SO_4_, 0 µM, 0.1 µM, 1 µM, 10 µM, 100 µM, 1 mM, and 10 mM Fe). Pure 500 mL ASW triplicates were set up as controls. After 7 days of incubation at 4 °C in the dark, the bottles were sampled for characterization of the indicated parameters.

To date, studies on hydrothermal plumes have either dealt with identifying the dominant microbial lineages and relating that with general chemistry^25^, assessing the genomic and/or transcriptomic potential^26^, describing and characterizing Fe-binding ligand concentrations/compositions, or measuring Fe-concentrations^27^. However, a holistic experimental approach combining microcosm experiments along Fe-gradients with spiked plume water samples, where microbial community changes are monitored along with evolving molecular characteristics of DOM and Fe-binding organic ligands, is still lacking. In this pilot study, we describe such a systematic experiment and evaluate how variable Fe-concentrations in an incubation solution using natural plume samples affect the structure of microbial vent communities, the composition of related DOM, and excess Fe-binding ligand concentrations.

## Results

In the following, we present the results of short-term incubation experiments (7 days) with hydrothermal plume waters from Brothers volcano (1549 m water depth, deep plume maximum; Kermadec arc, Pacific) along an Fe-gradient suitable to test the behavior of microbes at naturally occurring both low and high Fe-concentrations (one control treatment with pure artificial seawater (sterile control), one control treatment with no Fe addition, and six Fe-treatments; all in triplicates; see Figs. 1 and 2 and detailed description in the methodology). This experiment is the first to address the questions of microbially mediated Fe-binding ligand production using an interdisciplinary approach. We present a comprehensive dataset on Fe-concentrations, DOM bound to Fe and excess Fe-binding ligands, alongside shifts in transcript microbial community structure. We document a systematic variability within the samples after a 7-day incubation and discuss possible implications (see Tables 1, 2).

**Table 1:** Dissolved iron (dFe), iron-binding ligand excess (L′), dissolved organic carbon (DOC, SPE-DOC), total dissolved nitrogen (TDN, SPE-TDN) and variability in the microbial community* within the original plume sample and post incubation.

Sample	Fe	L′	DOC	SPE-DOC	TDN	SPE-TDN	Arcob	Sulf	Massil	Oleisp.	SUP05	low.ab
	[µmol L^−1^]	[µmol L^−1^]	[µmol L^−1^]	[µmol L^−1^]	[µmol L^−1^]	[µmol L^−1^]	[%]	[%]	[%]	[%]	[%]	[%]
Plume sample (P)	0.071	0.143	75	20	35.2	0.9	0.0	1.8	0.0	0.1	86.6	11.0
Initial solution 50:450 (P:C)**	0.275	0.041	790	9	450.5	0.6	–	–	–	–	–	–
**Post incubation samples**												
Control (C) I (*)	0.691	–	786	10	502.4	0.7	–	–	–	–	–	–
Control (C) II	0.295	0.029	849	8	489.5	0.3	–	–	–	–	–	–
Control (C) III	0.302	–	973	6	498.1	0.6	–	–	–	–	–	–
Inc 0 µM Fe I	0.225	0.196	368	24	4.2	1.2	2.6	60.9	0.2	1.7	32.5	2.0
Inc 0 µM Fe II	0.137	–	511	18	4.4	2.0	2.5	54.1	0.2	0.0	39.4	3.0
Inc 0 µM Fe III	0.257	–	577	28	5.3	1.2	0.1	90.8	0.0	1.8	6.4	1.0
Inc 0.1 µM Fe I	0.201	0.230	339	29	7.1	1.3	0.1	92.8	0.0	1.7	4.8	0.6
Inc 0.1 µM Fe II	0.149	–	420	26	2.4	1.0	0.0	56.7	0.0	6.0	35.1	2.2
Inc 0.1 µM Fe III (†)	0.144	–	471	34	2.9	6.7 (†)	1.2	90.5	0.0	2.2	5.8	0.3
Inc 1 µM Fe I	0.96	2.13	467	28	4.7	0.8	3.2	55.7	0.1	9.8	30.1	1.1
Inc 1 µM Fe II	1.14	–	318	23	2.7	1.3	1.7	80.2	0.0	4.2	13.6	0.3
Inc 1 µM Fe III	0.89	–	926	25	3.0	1.2	1.3	79.1	0.0	0.1	19.1	0.4
Inc 10 µM Fe I (†)	9.9	–	511	20	2.5	2.6 (†)	3.5	28.1	0.9	2.7	62.0	1.7
Inc 10 µM Fe II	10.0	–	671	28	4.0	1.0	0.3	83.2	0.2	0.0	15.9	0.5
Inc 10 µM Fe III	10.7	16.3	389	30	1.7	1.2	1.4	64.2	0.0	6.1	27.2	1.0
Inc 100 µM Fe I (†)	95.0	–	394	28	2.1	2.4 (†)	1.5	71.1	0.1	0.5	26.2	0.5
Inc 100 µM Fe II	107	132	677	25	2.9	1.3	5.7	47.6	0.1	1.9	43.4	1.2
Inc 100 µM Fe III	103	–	1030	27	4.1	1.2	1.1	75.3	0.0	0.2	22.7	0.7
Inc 1 mM Fe I (‡)	975	–	886	20	2.9	0.9	– (‡)	– (‡)	– (‡)	– (‡)	– (‡)	– (‡)
Inc 1 mM Fe II (‡)	1000	291	8842	23	52.3	1.2	– (‡)	– (‡)	– (‡)	– (‡)	– (‡)	– (‡)
Inc 10 mM Fe I	10,291	–	648	35	3.0	1.6	0.0	0.8	4.1	0.0	89.4	0.3
Inc 10 mM Fe II	10,273	–	668	28	2.5	1.1	0.0	0.6	2.6	0.0	92.3	0.3
Inc 10 mM Fe III	10,399	–	702	22	3.0	0.9	0.0	0.0	2.4	0.0	97.5	0.1

Arcob., Arcobacteraceae; Sulf., Sulfurimonas; Massil., Massilia; Oleisp., Oleispira; SUP05, SUP05 Gammaproteobacteria; low.ab., various, too low individual abundance.

†Excluded from the evaluation of DOM as SPE-TDN exceeds TDN indicating contamination.

‡Excluded as number of sequences too low for microbial community analysis and DOM of replicates is inconclusive (see Table 2).

*Excluded for calculation of 50:450 (P:C) initial solution as an outlier as both other replicates are in very good agreement.

**Values for the 50:450 (P:C) initial solution were calculated from plume and control end members.

**Table 2:** Average characteristics of solid-phase extracted dissolved organic matter (SPE-DOM) within the original plume sample and post incubation.

Sample	m/z	H/C	O/C	AI_mod_	DBE	NOSC	I_DEG_^¥^	#Form	D_F_(C)^○^	D_F_(H/C)^○^	D_F_(NOSC)^○^	#Fe–Form
	[–]	[–]	[–]	[–]	[–]	[–]	[–]	[–]	[–]	[–]	[–]	[–]
Plume sample (P)	407	1.28	0.45	0.237	8.13	− 0.34	0.76	1722	2.19	0.080	0.160	94
Initial solution 50:450 (P:C)**	297	1.40	0.34	0.243	5.83	− 0.66	0.29	–	2.54	0.162	0.246	–
**Post incubation samples**												
Control (C) I	280	1.40	0.31	0.255	5.57	–	–	436	–	–	–	–
Control (C) II	265	1.46	0.29	0.231	5.00	–	–	382	–	–	–	–
Control (C) III	268	1.43	0.30	0.247	5.23	–	–	365	–	–	–	–
Inc 0 µM Fe I	415	1.27	0.46	0.235	8.30	–	–	1820	–	–	–	–
Inc 0 µM Fe II	414	1.27	0.46	0.235	8.26	–	–	1801	–	–	–	–
Inc 0 µM Fe III	415	1.28	0.46	0.235	8.28	–	–	1810	–	–	–	–
Inc 0.1 µM Fe I	414	1.28	0.46	0.235	8.26	–	–	1809	–	–	–	–
Inc 0.1 µM Fe II	409	1.28	0.45	0.237	8.17	–	–	1747	–	–	–	–
Inc 0.1 µM Fe III (†)	373	1.29	0.42	0.257	7.61	–	–	1226	–	–	–	–
Inc 1 µM Fe I	398	1.28	0.44	0.242	8.04	–	–	1464	–	–	–	–
Inc 1 µM Fe II	396	1.28	0.44	0.243	8.00	–	–	1513	–	–	–	–
Inc 1 µM Fe III	393	1.30	0.42	0.244	7.88	–	–	1585	–	–	–	–
Inc 10 µM Fe I (†)	411	1.27	0.47	0.237	8.27	–	–	1771	–	–	–	–
Inc 10 µM Fe II	380	1.29	0.42	0.251	7.72	–	–	1334	–	–	–	–
Inc 10 µM Fe III	367	1.30	0.40	0.258	7.53	–	–	1218	–	–	–	–
Inc 100 µM Fe I (†)	354	1.30	0.39	0.265	7.33	–	–	1237	–	–	–	–
Inc 100 µM Fe II	386	1.29	0.43	0.248	7.83	–	–	1403	–	–	–	–
Inc 100 µM Fe III	379	1.29	0.42	0.251	7.72	–	–	1354	–	–	–	–
Inc 1 mM Fe I (‡)	411 (‡)	1.28	0.46	0.237	8.25	–	–	1799	–	–	–	–
Inc 1 mM Fe II (‡)	364 (‡)	1.31	0.39	0.258	7.47	–	–	1366	–	–	–	–
Inc 10 mM Fe I	355	1.31	0.39	0.263	7.34	–	–	1187	–	–	–	–
Inc 10 mM Fe II	361	1.31	0.39	0.261	7.43	–	–	1282	–	–	–	–
Inc 10 mM Fe III (•)	402 (‡)	1.28	0.45	0.240	8.07	–	–	1679	–	–	–	–
Averages for triplicates with DOM characteristics of the reduced dataset (section = only formulae occurring in all replicates)*												
Control (C) section	267	1.43	0.30	0.245	5.20	− 0.75	–	317	2.64	0.185	0.269	7
0 µM Fe section	414	1.27	0.46	0.235	8.28	− 0.31	0.80	1794	2.18	0.083	0.168	117
0.1 µM Fe section	411	1.28	0.46	0.236	8.22	− 0.32	0.79	1741	2.20	0.082	0.166	98
1 µM Fe section	394	1.28	0.44	0.244	7.97	− 0.38	0.74	1403	2.29	0.081	0.157	28
10 µM Fe section	372	1.30	0.41	0.255	7.60	− 0.45	0.67	1197	2.43	0.093	0.162	11
100 µM Fe section	381	1.29	0.42	0.250	7.76	− 0.42	0.69	1309	2.37	0.087	0.158	14
10 mM Fe section	357	1.30	0.39	0.263	7.38	− 0.50	0.61	1172	2.54	0.101	0.167	8
NEqPIW section (N = 5)	424	1.28	0.45	0.235	8.42	− 0.33	0.84	1527	2.30	0.079	0.158	–

Values for the 50:450 (P:C) initial solution were calculated from SPE-DOC weighed average characteristics (see Table 1). Average mass of formulae (m/z); elemental ratios (H/C, O/C); average modified aromaticity (AImod); double bond equivalent (DBE) after Koch and Dittmar (2006, 2017); North Equatorial Pacific Intermediate Water (NEqPIW)—in-house standard.

†Excluded from the evaluation of DOM as SPE-TDN exceeds TDN indicating contamination (see Table 1).

‡Excluded as number of sequences too low for microbial community analysis (see Table 1) and DOM of replicates is inconclusive.

•Excluded from further evaluation of DOM as an outlier as both other replicates are in very good agreement.

*Reduced dataset refers to further evaluation of DOM after exclusion of specific incubations (†, ‡ and •).

**Values for the 50:450 (P:C) initial solution were calculated from plume and control end members.

¥I_DEG_ of initial solution 50:450 (P:C) based on weighted intensities of control (9×) and plume sample (1×).

○Diversity indices used by Mentges et al.^42^; D_A_ Gini-Simpson index; D_F_(C, H/C, NOSC) Rao's entropy—functional diversity.

### Variations of dissolved Fe and Fe-binding ligands

Dissolved Fe-concentrations (dFe) of all samples are given in Table 1. The dFe concentration of the sampled hydrothermal plume (P) was quantified as 0.071 µM. The average [dFe] of the control samples (C), being the pure artificial seawater medium (ASW) at the experiment end on day 7, was 0.298 ± 0.005 µM (average of replicates II and III, replicate I—was neglected as an outlier). The elevated and variable [dFe] in the control is indicating that a systematic blank might be coming from the chemicals used to prepare the ASW, and/or that some contamination especially in the excluded Control replicate I might have been introduced via sample handling. Only negligible amounts of complementary DNA (cDNA) sequences were detected in the controls, and it can therefore be assumed that no microbially induced changes occurred, just as no significant abiotic changes can be expected under the selected incubation conditions. Since the initial incubation solution at T_0_ is based on the mixing ratio of the plume with the control sample at a volume ratio (P:C 50:450 mL) a [dFe] of 0.275 ± 0.005 µM Fe is calculated. There is a good recovery of the added nominal Fe at the end of the incubation, measured as dFe (< 10% deviations for > 1 µM Fe). Only the two incubations with the least Fe-amendment (0 and 0.1 µM Fe), exhibit final Fe-concentrations on average 33% below the ones anticipated for the spiked starting mixture and this might reflect a limited, potentially microbial depletion, a loss due to adsorption on small-sized particles or the container wall^28^, or simply an overestimation of the average dFe in the initial solution.

**Figure 3:**
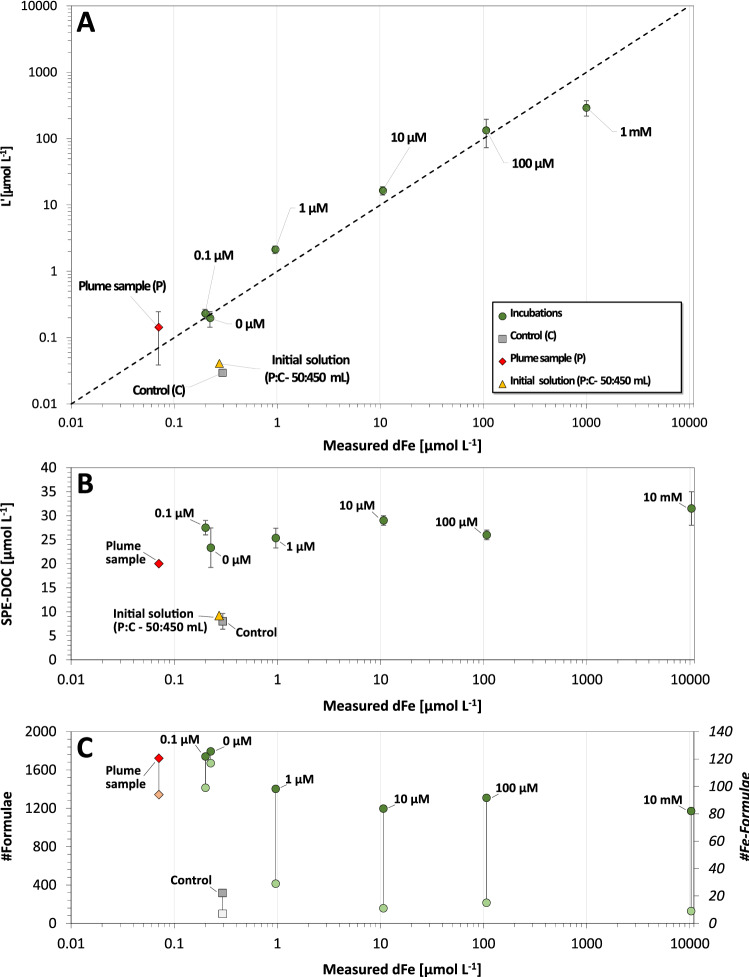
(**A**) Measured dissolved Fe (dFe) versus excess ligand concentrations (L′) in the different incubations including the original plume sample. The initial solution was calculated from values of the original plume and the pure ASW. (**B**) Final average SPE-DOC concentrations in the different incubation sets. (**C**) Total number of different formulae identified in the SPE-DOM isolated from the different samples. The connected second, light colored symbol for each sample refers specifically to the number of Fe-containing formulae.

Data on Fe-binding ligands show that all samples had an excess of ligands (L′), with [L′] = [L] − [dFe], and [L] being the initially derived Fe-binding ligand concentration (see Supplemental Table S5). The excess of ligands increased with increasing Fe in the incubations (see Table 1 and Fig. 3A). This ligand excess indicates a passive (lysis) or an active microbial production of additional Fe-binding ligands over the 7-day incubation period. A certain excess production of ligands can be expected for an attempt to quickly neutralize potentially toxic levels of dFe. Possibly, microbes initially present in the 1 mM Fe-incubations did not manage to effectively neutralize Fe and were eventually overwhelmed. Accordingly, amounts of RNA detected in the samples were insufficient for a microbial community analysis. Ligand data could not be derived for the 10 mM sample, as high dilution factor required would result in an error > 100% and the formation of Fe-hydroxide in the oxidized sample would interfere with voltammetric analysis (see Supplementary Results S1 for details on the implications in context of ligand stabilities).

### Variations in dissolved organic carbon (DOC) and total dissolved nitrogen (TDN)

In addition to DOC and TDN within the bulk sample, solid-phase extractable shares (SPE-DOC and SPE-TDN) were quantified for SPE-DOM^29^. Measured DOC was high in all incubations including the control replicates indicating that the ASW contained a considerable contamination of supposedly rather simple organics (probably derived from the chemicals used for the preparation; see Table 1) and hence this data is considered inconclusive. In contrast to bulk DOM which also includes very volatile, low molecular weight organic compounds, SPE-DOM is much less prone to contamination. In contrast to total DOC, SPE-DOC was exceptionally low in the controls (and thus also in the derived initial solution) but later experienced a considerable 3–fourfold increase in all incubations (see Fig. 3B and Table 1). While we observe no clear trend between the different incubation sets, the slight SPE-DOC increase between 0 and 10 mM Fe could be interpreted as microbial production of more complex solid-phase extractable SPE-DOM compounds in unfavorable Fe conditions. High levels of TDN in the controls can be subjected to NH_4_Cl used for the preparation of the ASW. Reasons for the close to quantitative but uniform loss of TDN from all incubations are discussed in more detail below in the context of the documented prevalent microbial groups.

### Variations in microbial community structure along the Fe-gradient

The RNA levels in the replicates of the sterile control were below the threshold of 1000 merged reads, indicating that they are free of microbial contamination and thus the controls can be treated as true controls. 16S tag profiling based on DNA from the plume and RNA from the incubation microcosm experiments demonstrated shifts in the microbial communities along the Fe-gradient. The DNA-based 16S tags from the original plume, identifies both living and dead cells. The RNA-based 16S tag analyses from the incubations along the Fe-gradient, reflects only active parts of the microbial community likely best adapted to the given experimental conditions. Given the very low biomass of extracted archaeal RNA, the taxonomy results are only briefly presented (Supplementary Fig. S1 and Results S2). The original hydrothermal plume habitat was dominated by the SUP05 clade of Gammaproteobacteria (87%) with a minor contribution of *Sulfurimonas* (Campylobacterota) (2%) and a range of not further distinguished taxa with very low individual abundances (in total representing approx. 11%). Representatives of both the *Sulfurimonas* genus and the SUP05 clade have been frequently encountered in suboxic and anoxic waters enriched in sulfide including hydrothermal fluid and plume environments^25,26,30^. Their success in redox zones has been suggested to be related to them facilitating lifestyles as k- and r-strategists^31^. *Sulfurimonas* spp. exhibit a very broad metabolic flexibility^30,32^. They can grow autotrophically using sulfur compounds and hydrogen as an electron donor and oxygen or nitrate, nitrite, particulate manganese oxide as well as various organic compounds as an electron acceptor^30,32,33^. Physiological characterization of cultivated representatives of the widespread SUP05 group showed that, in addition to autotrophic carbon fixation fueled by sulfur-oxidation, chemoorganic heterotrophic lifestyles may also be present^34^. Both, SUP05 and Sulfurimonas, have been confirmed to harvest energy from oxidation of reduced compounds (sulfides) or elemental S. SUP05 is not known to use SO_4_^2−^ as an electron acceptor. One study reports that Sulfurimonas has the genetic make up for a coupled reduction of SO_4_ using H_2_ as a reducing agent, but the energy yield of that route is low compared to others like nitrate reduction or thiosulfate oxidation and to date no study has provided evidence of the actual realization of that route^33^. Hence, sulfate should not have a major impact in the incubation medium for fueling growth of SUP05 or Sulfurimonas. Under anaerobic conditions, SUP05 has been demonstrated to be capable of converting ammonium (NH_4_^+^) to nitrite (NO_2_^−^). In conjunction with Sulfurimonas’ ability to use nitrite in autotrophic synthesis of organic compounds, the quantitative loss of TDN from all incubations is not unexpected.

**Figure 4:**
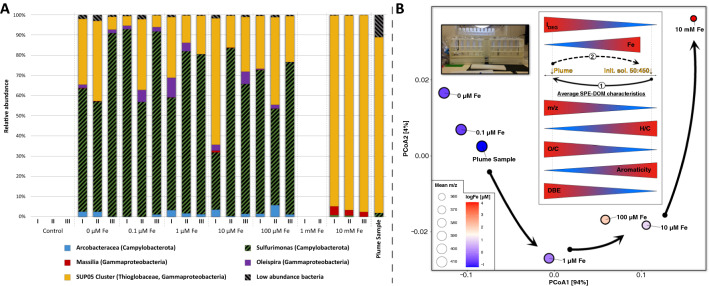
(**A**) Taxonomy plots of the bacterial communities within the incubations after 7 days. Only plots with a minimum of 1000 merged sequences per sample are shown. (**B**) Principal coordinate analysis depicting the variability of SPE-DOM composition in the incubation experiments. Average SPE-DOM compositions were calculated for the different levels of spiked Fe. The embedded sketch depicts the incubations plot with respect to selected average SPE-DOM characteristics (m/z, H/C, O/C, AI_mod_ and DBE as well as the degradation index I_DEG_^37^) relative to the original plume sample and the composition of the initial solution (50:450 (P:C) medium to plume sample ratio) mixture. Path number 1 indicates the general change presumably associated with microbial growth, whereas the reverse path number 2 will in part be associated with DOM-Fe-coagulation.

Relative to the natural plume habitat, the microbial community compositions shifted in the incubation experiments amended with different Fe-concentrations (see Fig. 4A and Table 1), although the bacterial 10 mM Fe incubation communities were more closely related to those found in the natural plume sample rather than to those of the other Fe amended incubations (see Supplementary Fig. S2). Based on RNA-profiling the incubations with low to intermediate Fe-concentrations (0–100 µM Fe) were dominated by *Sulfurimonas* (Campylobacterota) (48–93%) and members of the clade SUP05 (Gammaproteobacteria). No obvious trends for the shifts in *Sulfurimonas* or SUP05 proportions were visible in 1–100 µM Fe-supplemented experiments. However, at the highest Fe-concentration (10 mM), *Sulfurimonas* (< 1%) was completely outcompeted by the SUP05 clade (89–97%). This shift might point to missing or insufficient strategies by *Sulfurimonas* for dealing with elevated Fe-levels, i.e. avoiding cell encrustation through production of Fe-binding ligands that keep Fe in solution. The share of SUP05 even increased significantly (*p* ≤ 0.05) from the original plume sample, indicating that these bacteria are well adapted to dealing with enriched Fe-concentrations. Intriguingly, albeit *Sulfurimonas* and SUP05 are dominant affiliates in plume and other oxygen-poor waters, to date, they have not been directly associated with Fe-binding ligand production. Yet, a recent gene expression study suggested that the affiliates of the uncultured SUP05 have the genetic potential to oxidize Fe^35^.

While clearly subordinate to the dominant SUP05 clade, a second group of Gammaproteobacteria of the genus *Massilia* (2.4–4.1%) are active in the 10 mM Fe-incubations. It has previously been shown that the *Massilia* sp. NR4-1 representative reacts to Fe-deficient conditions with the production of the siderophore massiliachelin likely to counteract the Fe-limitation^36^. However, a SPE-DOM formula corresponding to the massiliachelin formula was not detected in our experiments. Yet, in our experiments a different *Massilia* strain was detected and the tendency of this genus to react to Fe-limiting conditions by producing complexing agents suggests that our strain may also have the capability to produce Fe-binding compounds in Fe-replete conditions. At the low to intermediate Fe-concentration levels (0–100 µM Fe), the Gammaproteobacterium *Oleispira* and some uncultured *Arcobacteracea* complement the microbiome.

### Changes in composition of dissolved organics in relation to microbiology, and Fe systematics

The composition of SPE-DOM within the incubations showed a systematic variation with the Fe-concentrations (see Fig. 4B and Table 2). Reference for the direction of the changes is again the reconstructed SPE-DOM deficient initial solution (SPE-DOC = 9 µM) and comprises mostly oxygen poor (O/C = 0.34, even 98% oxygen poor-formulae in the pure medium), comparably saturated (H/C = 1.40, DBE = 5.83) compounds of low average mass (m/z = 297). Considering the exclusive SPE-DOC increase in all non-sterile incubations, the major share of the final SPE-DOM was thus presumably microbially produced during the incubation. All incubations systematically shifted towards a more oxygenated composition with a higher degree of unsaturation and an average mass closer to the original plume sample (O/C = 0.45, H/C = 1.28, DBE = 8.13 and m/z = 407) and this shift becomes more limited with increasing Fe-concentration (see Fig. 4B).

For each Fe-level, we calculated values for a degradation index (I_DEG_) to assess to what extent compositional variability could result from differing degrees of microbial production and turnover (see Table 2)^37–39^. For the initial solution, I_DEG_ indicates the most undegraded SPE-DOM composition (I_DEG_ = 0.29). The cultivation experiments then systematically shift towards a more degraded SPE-DOM (see Fig. 4B). The low Fe-incubations (0 and 0.1 µM Fe; I_DEG_ ~ 0.79) end up with a similar degradation level as the original plume sample (I_DEG_ = 0.76; see Table 2). Incubations with more Fe (1–100 µM Fe) appear less degraded (I_DEG_ = 0.69–0.74) with the 10 mM Fe-incubation exhibiting the lowest value (I_DEG_ = 0.61). Despite the deviation of incubation and in-situ conditions, the plume sample microbes might therefore produce an SPE-DOM mixture that approximates the composition found in the natural habitat and the magnitude of microbial growth and DOM reworking is simply higher at low Fe-concentrations (path 1 in Fig. 4B). Then again, there is no particular trend in the amount of produced SPE-DOC. Therefore, microbes prevailing at lower Fe-concentrations might produce DOM compositions that deviate more from the original SPE-DOM. But the microbial community structure is indistinguishable between 0 and 100 µM Fe and only clearly differs for the highest 10 mM Fe-level. The SPE-DOM variability could hence be additionally linked to other factors like a selective coagulation of certain SPE-DOM fractions with Fe^40^. And in fact, the apparently more limited change at high Fe-levels would be in line with a previously documented preferential scavenging of larger (high m/z), oxygen-rich (high O/C) and rather unsaturated (low H/C and high DBE) compounds by Fe-coagulation (see path 2 in Fig. 4B)^41^. Therefore, SPE-DOM composition might be subjectable to both microbial production and reworking and selective coagulation that increases with Fe-availability. To evaluate how these factor contributed, we looked at additional SPE-DOM characteristics and different diversity indices (see Table 2 and detailed definition in Mentges et al.^42^). The most diverse SPE-DOM with respect to number of individual formulae is held by low Fe-incubations (1800) and decreases with increasing Fe until only ~ 1200 formulae remain in the 10 mM Fe-incubation set (see Fig. 3C). This trend seems to point to a progressive reduction in molecular richness through Fe-coagulation rather than to a decreased diversity of the microbial community, except for the highest Fe-concentrations (i.e., clear dominance of SUP05)^40,41^.

In terms of functional diversity indices, those measuring reactivity (D_F_(C)) and bioavailability (D_F_(H/C)) both decrease together with Fe. This decrease is in agreement with the low Fe-incubation supposedly having experienced a higher extent of microbial degradation (higher I_DEG_). In contrast, the D_F_(NOSC) (oxidation of high NOSC compounds should be more profitable for microbes) increases with decreasing Fe and increasing I_DEG_, but the opposite should be true^42^. Changes in NOSC, hence, rather point to Fe-coagulation than microbial degradation^40^. However, the highest 10 mM Fe-incubation holds a comparably high D_F_(NOSC) and this could be linked to the clear SUP05 dominance. Previous studies investigating coagulation for terrigenous DOM observed a preferential removal of aromatic compounds but in our cultivation experiments aromaticity is increasing with Fe (see Table 2 and Fig. 4B). Thus, hypothetically, microbes in this study produced less aromatic SPE-DOM in the lower Fe-treatments (higher I_DEG_, lower AI_mod_) and in particular aromatic SPE-DOM in the highest Fe-treatments that largely contained the SUP05 clade.

The described molecular diversity pattern also reappears with regards to 128 Fe-containing formulae identified over the entire dataset (see Fig. 3C, Table 2 and Supplementary Table S2). The undiluted plume sample contained a similar number as the 0 and 0.1 µM Fe-incubation sets (94 versus 117 and 98). This diversity rapidly decreases with increasing Fe-concentrations, with the 1 µM Fe-level already being down to 28 distinct Fe-formulae. Very few Fe-formulae persist at the higher 10 µM, 100 µM and 10 mM Fe-concentrations (11, 14 and 8). From high to low Fe, virtually all detected formulae on the higher levels are also found at the following lower levels^1^. The observed higher number of Fe-formulae may suggest that microbes only need to resort to versatile metabolic strategies and a range of different ligands to scavenge the little Fe available in their habitats when Fe concentrations are exceptionally low. Alternatively, if Fe reaches toxic levels, there is a shift in the metabolism to quickly produce sufficiently high ligand numbers to prevent encrustation and then quantity is prioritized over diversity. Then again, the reduced diversity of Fe-containing formulae could also be explained by the described Fe-DOM-coagulation.

**Figure 5:**
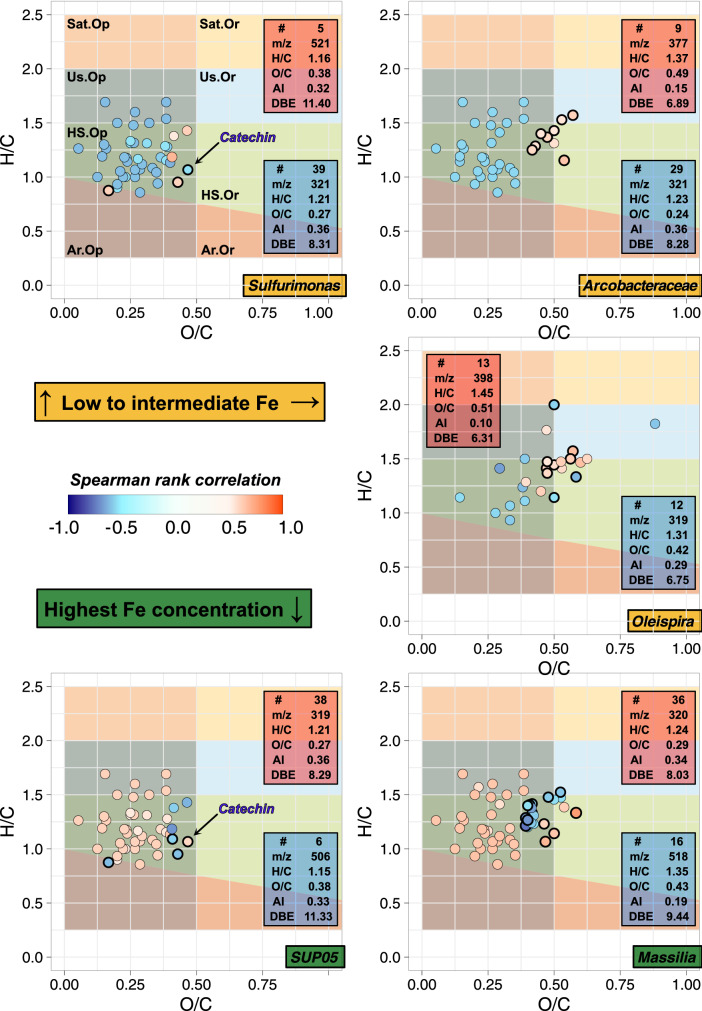
Van Krevelen plots depicting formulae that show a significant positive/negative correlation (*p* < 0.05) with the relative abundance of specific microorganisms, i.e., *Sulfurimonas*, *Arcobacteraceae*, *Oleispira*, *SUP05* and *Massilia*. Strength of the Spearman rank correlation indicated by color bar (only r > 0.5 | r <  − 0.5). Only the highlighted formulae (open black circles) are not also corelated with Fe. Colored areas indicate organic compound groups as defined in^53,73^. To maximize comparability, all sample sets were limited to the 1000 most abundant formulae and total intensity normalized to 1. Average characteristics of formulae for which relative intensity correlates positively (red) and negatively (blue) with relative abundance of different microbial groups provided in insets. Abbreviations refer to number of formulae (#), average mass of the respective formulae (m/z), elemental ratios (H/C and O/C), aromaticity (AI) and double bond equivalent (DBE). Definitions of the latter are given in methods.

We further investigated if specific correlations exist between certain formulae within the SPE-DOM mixture and the relative abundance of specific microorganisms (see Fig. 5 and Supplementary Table S3). Most formulae significantly correlated with a group (Spearman rank correlation, *p* < 0.05) plot within the field of highly unsaturated or unsaturated O-poor components. A considerable overlap exists between *Sulfurimonas* plus *Arcobacteraceae* (rather negative correlation) and the SUP05 clade plus *Massilia* (rather positive correlation). This overlap can mostly be explained with Fe-coagulation (relative increase of O-poor formulae is expected as O-rich formulae are preferentially removed) as the latter clearly dominate the 10 mM Fe-incubation set. Only few formulae do not also correlate with Fe-concentration and appear specifically linked to the abundance of distinct microbial groups (see highlighted formulae in Fig. 5). Most noteworthy is a formula corresponding to the phenolic ligand Catechin (C_15_H_14_O_6_) that is known for its potential to complex Fe but as no structural information is available it cannot be evaluated if this isomer is actually present^43^. Only 5 Fe-containing formulae are significantly correlated (Pearson correlation, *p* < 0.05) with one of the 5 identified main microbial groups (see Supplementary Tables S4.1,  S4.2). Then again, all abundances are also correlated with Fe and with its mostly negative correlation it is unlikely that these compounds are produced by the respective microbial group. Nevertheless, C_20_H_23_FeN_3_O_2_-m/z 392, C_24_H_33_FeN_3_O_4_-m/z 482 and C_22_H_38_FeO_7_S-m/z 501 are positively correlated with *Sulfurimonas* and C_17_H_30_FeN_6_O_8_-m/z 501 with *Oleispira* and this could mean that their presence is linked to the activity of these groups.

## Discussion

This pilot experimental study provides first insights into how plume microbial communities might respond to variable Fe-enriched environments. Microbial community structure was assessed in conjunction with Fe, Fe-binding ligands, and SPE-DOC levels, as well as the SPE-DOM composition along a broad Fe-gradient. The plume microbes, dominated by some Campylobacterota (*Sulfurimonas*) and Gammaproteobacteria (SUP05 clade), react to Fe-addition possibly by producing organic complexes, i.e. ligands, however, a small proportion of passively released Fe-binding ligands from virus induced cell lysis cannot be excluded^27,44^. The excess of ligands found in each sample might point to microbial strategies for managing Fe-availability in low-Fe conditions and avoiding cell encrustation in Fe-replete conditions which allows these plume microbes to survive in dynamic habitats with various Fe-gradients such is the case for hydrothermal plumes or vents^2,3,11^.

While a mixture of *Sulfurimonas* and SUP05 clade (± *Arcobacteraceae*, *Oleispira* and *Massilia*) coexist at low to intermediate Fe-levels (0–100 µM), in the extreme 10 mM Fe-amended incubations, the bacterial community clearly shifted to include a dominance (~ 93%) of SUP05 (± *Massilia*) affiliates (see Fig. 4A). This shift is in line with another recent study finding that different plume adapted specialized Gammaproteobacteria of SUP05 clade are characterized by a high expression of heavy-metal resistance (e.g., for avoiding cell encrustation) and Fe-acquisition genes and hence might be exclusively capable of dealing with such high Fe-levels^35^. The clear dominance of SUP05 in the original plume sample despite it’s relatively low Fe contents (0.072 µM) could be interpreted to mean that most of the microbes in the plume were actually exported from near the vent, where mM Fe concentrations levels are frequently encountered in the dynamic environment at Brothers^45,46^. In addition, the anoxic conditions set in the incubations (H_2_:CO_2_ purging of the solution and replacement of the bottle headspace) could have favored *Sulfurimonas* compared to the more oxygenated environment of the plume (> > 150 µmol/kg O_2_). While both bacteria are found in similar environments (suboxic, anoxic and sulfidic), *Sulfurimonas* was found to dominate over SUP05 under more anoxic conditions (anoxic and sulfidic)^31^. While the total SPE-DOC concentrations increase is rather indistinguishable over all incubations, the SPE-DOM degradation index I_DEG_ indicates a considerably more degraded DOM composition at lower Fe-concentrations (see Figs. 3B, 4B and Table 2). It is likely that as Fe content increased, the microbes had to produce more and more ligands to avoid cell encrustation and overall microbial growth was inhibited, ultimately resulting in a less diverse SPE-DOM composition. Further support for this hypothesis is provided by evaluation of Fe-containing formulae within the SPE-DOM mixture (see Fig. 3C). A considerably higher diversity of individual Fe-formulae (~ 100) in the low-Fe-incubations (0 and 0.1 µM) may reflect a more versatile microbial community structure. At higher Fe-concentrations (> 10 µM) only very few Fe-containing formulae remain (~ 10) and most of the ones with N-heteroatoms disappear (see Supplemental Table S2). Synthesizing compounds with heteroatoms requires more effort and thus the absence of these compounds would be in line with a greater stress level caused by toxic Fe-concentrations. Simultaneously, part of the SPE-DOM compositional variability clearly appears subjectable to a selective DOM-Fe-coagulation that increases with Fe-concentration. The nature of the related changes is mostly in agreement with previous findings for Fe-coprecipitation with more terrigenous DOM (preferential removal of larger oxygen-rich unsaturated high NOSC compounds)^40^. But in contrast to these findings, SPE-DOM aromaticity increases with Fe and the bimodal split (with SUP05 and Massilia clades dominating at 10 mM Fe) is reflected in the reactivity related functional diversity index D_F_(NOSC). Overall, SPE-DOM variability depicts noticeable trends that are in agreement with broader microbial activity as well as the selective DOM-Fe-coagulation, and even suggests a relationship between specific microorganisms and certain formulae (Fe- and non-Fe-containing). In conjunction with Fe, ligand and microbial systematics, the broad range of parameters monitored in this pilot experimental study delivered a multitude of new potential implications regarding the significance of Fe in a hydrothermal plume environment. Future targeted experiments are needed to unequivocally differentiate and quantify the feedbacks between microbial community, dissolved organics including organic ligands, abiotic ligands and Fe-organic coagulation under different Fe-enriched conditions.

## Methods

### Working area and sample collection

A large volume water sample was acquired from a buoyant hydrothermal plume at Brothers volcano during an expedition with R/V Sonne (SO253 in 2016/2017) to the Kermadec arc in the South West Pacific. This region is characterized by pronounced volcanic activity in context of a tectonic trench-ridge setting in which the Pacific plate is subducted under the Australian plate, with Brothers volcano being the best studied system at the Kermadec arc^45^. Brothers volcano is a 3 km wide caldera volcano with active venting present at the NW Caldera Wall, the Upper Caldera and two resurgent cones^46^. Hydrothermal fluids from the NW Caldera Wall yield high Fe-concentrations of up to 12.4 mM^46^ and together with an extensive plume spreading (up to 12 km) with Fe-concentrations up to about 200 nM within the caldera^47^, Brothers volcano is the perfect environment for our process-oriented study. The plume at Brothers was sampled with an SBE32 carousel water sampler (22× 10L NISKIN bottles) coupled to a conductivity temperature depth (CTD) unit (SBE911plus). Plume sample *053 CTD 04* was taken in 1549 m water depth within the deep plume maximum (34° 51.733’ S | 179° 3.559’ W) as indicated by optical backscatter (Seapoint Turbidity Meters), potential temperature and oxidation reduction potential (PMEL) anomalies. The plume sample can be expected to contain a diverse selection of deep-sea and especially hydrothermal vent adapted microbes.

### Set-up of incubation experiments and sampling

For the experimental investigation, different incubations were set up with an ASW (MJ-medium consisting of 30 g NaCl, 0.14 g K_2_HPO_4_, 0.14 g CaCl_2_·2H_2_O, 0.25 g NH_4_Cl, 3.4 g MgSO_4_·7H_2_O, 4,18 g MgCl_2_·6H_2_O, 0.33 g KCl, 0.01 g, 0.5 mg NiCl_2_·6H_2_O, 0.5 mg Na_2_SeO_3_·5H_2_O per liter)^48^. The control incubations consisted of 500 mL MJ-medium without Fe additions (Control). Regular incubations were set up with 450 mL MJ-medium and 50 mL plume (*053 CTD 04*) water and a range of different Fe(II)SO_4_ concentrations (0 µM, 0.1 µM, 1 µM, 10 µM, 100 µM, 1 mM and 10 mM, Table 1). The plume incubations were performed in acid-cleaned polyethylene bottles and purged with H_2_:CO_2_ (80:20). Triplicates for all Fe-concentration levels were incubated for 7 days at 4 °C in the dark. 7 days incubation time was set as a compromise between integrating slower growing microbes and accounting for the bottle effect likely caused by longer incubation times. After incubation, the whole sample was filtered through 0.2 µm GTTP Polycarbonate filters (Merck Millipore Ltd.) to concentrate cell material on the filters and to collect the filtrate for further chemical analysis. The filters were immediately stored at − 80 °C for later RNA analyses of 16S rRNA tags (see below). The filtrate of each plume incubation was then sub-sampled for quantification of total dissolved Fe (dFe), Fe-binding ligands, DOM, DOC, and TDN. For the analysis of dissolved Fe (dFe) the 50 mL of filtrate were transferred into acid-cleaned fluorinated high-density polyethylene (HDPE) bottles and immediately acidified using quartz distilled HCl (q-HCl) to pH < 2. Fe-binding ligand samples (250 mL) were transferred into acid cleaned HDPE bottles and frozen (− 20 °C) directly. For the combined quantification of DOC and TDN, 10 mL of the filtered sample were transferred into pre-combusted glass vials, acidified to pH 2 (ultrapure HCl) and stored at 4 °C in the dark. Another 75 mL of the filtrate was acidified to pH 2 (ultrapure HCl) and subjected to a solid phase extraction procedure to acquire a methanolic extract for the molecular characterization of the solid phase extractable fraction of DOM (SPE-DOM)^29^. In brief, the entire 75 mL were run through a styrene divinyl benzene cartridge (100 mg, PPL, Varian, pre-cleaned with methanol and conditioned with acidified ultrapure water) that adsorbs a representative fraction of DOM. Subsequently, the salt matrix was removed through repeated rinsing with acidified ultrapure water prior to drying the cartridge with N_2_(g) and ultimately recovering a SPE-DOM extract through elution with 1 mL of methanol (stored in a pre-combusted and pre-weight brown glass vial at − 20 °C).

### Limitation of the experimental design

To our knowledge, these are the first incubation experiments ever conducted with hydrothermal plume material addressing microbial reactions, i.e. community shifts and production of metabolites, as a reaction to Fe-amendment. The data provide an interesting first look into the interdependencies between Fe, dissolved organics, Fe-binding ligands and microbial community structure related to a hydrothermal plume environment. However, as these were the first experiments conducted, the study was designed as a proof-of-concept and has its limitations, which are briefly discussed in the following: The incubation experiments simulate the dilution of plume material during its non-buoyant mixing. In our case we chose artificial seawater (ASW) under a (80:20 H_2_:CO_2_) environment as the incubation medium. While this does not fully mimic the natural plume environment in the deep-sea it has the advantage that the medium provides optimal conditions for the natural variety of microbial clades present in an Fe-rich hydrothermal plume, to thrive. E.g. ammonium and phosphate are available in excess, and in addition, Hydrogen a common energy source for many microbial clades including *Sulfurimonas* and SUP05 was provided^30,49^. The medium initially also contained a considerable amount of DOC. While the identity of these organics is not defined, they are small labile molecules, which are not extracted by SPE. Extractable more complex compounds were virtually absent.

Overall, the presented incubation experiments demonstrate how the microbial community from the original plume behaves for variable Fe-enrichments in our particular growth medium under provided experimental conditions. Although these incubations are the first experiments showing shifts in the transcripts of the microbial community along an Fe-gradient, we acknowledge that the experiment would be much stronger would we have subsampled on in time points throughout the incubation, not only at the end. Reasons for this omission are (i) the experiment was ran as proof of concept, (ii) volumes incubated did not allow for sampling for all parameters at multiple time points, (iii) subsampling may have disturbed the incubation. Further, since neither the FT-ICR-MS based characterization of SPE-DOM nor the CLE-CSV Fe-binding ligands analysis provide structural information and therefore the data does not allow to draw an explicit link to potentially unique metabolites that are indicative of the dominant microbial clades documented in this study. Future studies should consider including additional targeted analysis (Nuclear Magnetic Resonance spectroscopy (NMR), Fluorescent Dissolved Organic Matter (FDOM)) that look for such metabolites.

### Determination of dFe and Fe-binding ligands

Dissolved Fe (dFe) was determined on diluted acidified samples at the Trace Element Centre of the University of Otago (New Zealand) by an Agilent 7500 ce quadrupole inductively coupled plasma-mass spectrometer (q-ICP-MS) equipped with an octopole collision cell and an autosampler^50–52^. Standard solutions for external calibration were prepared via a serial dilution of a SPEX CertiPrep multi-element standard (NIST traceable) in 2% v/v quartz distilled nitric acid (q-HNO3) to matrix-match standards and sample solutions. For quality control, an over-all method blank (Milli Q) and a certified reference material (NASS-5, National Research Council, Canada) were analyzed multiple times with the samples. The accuracy of the NASS-5 reference material spiked with 35.0 (reference value 4.01 ± 0.70 nM; measured value (n = 6) 35.7 ± 0.81 nM) was acceptable (precision < 3%) and the procedural blank showed no analytically significant Fe-level (< 2.69 nM; detection limit) for this study, thereby confirming the suitability of the method to accurately determine dFe in the high-Fe-samples. Finally, samples were dilution and blank corrected.

For the determination of Fe-binding ligands, the Competing Ligand Equilibration-Adsorptive Cathodic Stripping voltammetry (CLE-AdCSV) protocol of Kleint et al.^23^ investigating organic Fe-binding ligands in diverse hydrothermal vent environments, was followed. Due to the high Fe concentrations in the incubations, samples were diluted down to ~ 20 nM dFe with a NaCl solution of the same salinity as the sample (34.5 ‰, prepared with ultrapure deionized water and NaCl (ROTI ®METIC 99.999%, Roth)) to avoid the precipitation of Fe-hydroxides^23^. Fe-standard-solutions (using a 1000 ppm Fe single element standard, Joint Ventures), 1 mM salicylaldoxime (SA; Sigma Aldrich) standard solution, and borate buffer were prepared and used as described in Kleint et al. ^23^. The titration was prepared adding borate buffer with a final concentration of 10 mM, increasing Fe-concentrations to up to 100 nM Fe, and a final SA concentration of 25 μM. Sample aliquots were allowed to equilibrate overnight at room temperature (20 °C) and subsequently analyzed the next day in a trace metal clean laboratory at the Jacobs University, Bremen, Germany using a 797 VA Computrace (Methrom) with a three-electrode configuration, including a hanging mercury drop electrode (HMDE) as working electrode, a double junction Ag/AgCl/3 M KCl reference electrode and a glassy carbon counter electrode. The CLE-AdCSV method with SA as the competing ligand for Fe-binding natural ligands in seawater is only applicable for Fe(III) concentrations below approx. 100 nM, otherwise the assumptions (e.g. constant free SA concentration throughout the titration) for deriving the complexation parameters (ligand concentrations, L_i_ and conditional stability constants, logK_i_^cond^) no longer apply. Given the high dFe concentrations in the incubations, the resulting high dilution factors and associated uncertainties introduce considerable uncertainty in the complexation parameters, this is particularly the case for the 10 mM dFe treatment. Thus, the excess of Fe-binding ligand concentrations over dFe (Table 2) are therefore given only as qualitative, exploratory indications for the apparent production of Fe-binding ligands in response to increasing dFe-concentrations.

### Quantification of DOC, SPE-DOC, TDN and SPE-TDN

Concentrations of DOC and TDN were determined on a Shimadzu TOC-V_CHP_ analyzer via high temperature catalytic combustion. Accuracy (< 2.7% [DOC] and < 0.2% [TDN] deviation from expected value), precision (RSD%, < 4.2% [DOC] and < 2.1% [TDN]) and LOD (5 µM [DOC] and 5.8 µmol L^−1^ [TDN]) were monitored through repeated measurements of ultrapure water and an in-house deep-water reference material (Hansell, Florida Strait, 700 m water depth, batch No. 9, 2009, 44 µmol DOC L^−1^). For determination SPE-DOC and SPE-TDN, 100 µL of the methanolic extract were evaporated at 50 °C and re-dissolved in 10 mL of 0.01 M HCl prior to the analysis. Here, accuracy (< 3.3% [DOC] and < 1.3% [TDN]), precision (RSD%, < 4.1% [DOC] and < 5.3% [TDN]) and LOD (5.8 µmol L^−1^ [DOC] and 3 µmol L^−1^ [TDN]) for the respective analysis refer to the measured solutions. The actual SPE-DOC and SPE-TDN concentrations and errors were reconstructed considering the volume ratio of methanolic extract (~ 0.7 mL) to extracted sample (~ 75 mL) as well as the dilution through evaporation and re-dissolution of an extract aliquot prior to the analysis. In three individual incubations, namely one 0.1 µM Fe 3, 10 µM Fe 1 and 100 µM Fe 1, the measured SPE-TDN concentration exceeded TDN, indicating contamination and thus these samples were excluded from the evaluation of SPE-DOM on a molecular level (see indication in Tables 1, 2).

### Characterization of SPE-DOM

A detailed molecular characterization of SPE-DOM was done based on ultra-high resolution mass spectra acquired through analyses with a 15 Tesla SolariX XR FT-ICR-MS (Bruker Daltonics). An aliquot of the methanolic extracts was diluted with ultrapure water to prepare a 1:1 mixture with a SPE-DOC concentration of 5 mg C L^−1^. Following a filtration (0.2 µm, PTFE syringe filter) this solution was injected at a constant flow rate (2 µL min^−1^) and subjected to electrospray ionization (ESI, Bruker Apollo II, negative ionization mode, 4 kV). Mass spectra over a pre-defined m/z range (50–1000 Da) were recorded following ion accumulation over 0.1 s and combination of 175 individual scans. Replicated measurements were calibrated individually with the Bruker Daltonics Data Analysis software using an internal calibration list derived from an inhouse SPE-DOM reference standard (NEqPIW, North Equatorial Pacific Intermediate Water). Peaks in the calibrated spectra were identified and exported with a dedicated VBA script. The individual spectra were further processed using the freely available ICBM-OCEAN software^53^. In detail, a method detection limit (MDL)^54^ was applied (mean + 2SD) and systematic errors along the mass axis were identified and corrected through a nonparametric smoothing model in a recalibration step. Ultimately, all spectra were combined in one table (.csv) and molecular formulas from all possible combinations of C_1-100_, H_1-200_, O_1-100_, N_0-6_, S_0-2_, P_0-1_, Cl_0-2_ and Fe^+2/+3^_0–2_ were assigned to the peaks (m/z) with a mass error of < 0.5 ppm. By taking homologous series networks (CH_2_, CO_2_) into account and prohibiting combinations including NSP, N_2_S, N_3_S, N_4_S, N_2_P, N_3_P, N_4_P, NS_2_, N_2_S_2_, N_3_S_2_, N_4_S_2_, S_2_P and those with 3 or more heteroatoms (N, S, P) except for N_3-6_, Cl and Fe the routine efficiently removed unlikely double assignments. Further reduction of the number of possible formulas was achieved by C isotope verification. The resulting cross table was complemented by elemental counts (C, H, O, N, S and P), ratios (H/C, O/C) and various indices including a double bond equivalent (*DBE* = 1 + 0.5 · (2*C* − *H* + *N* + *P*)), an aromaticity index (*AI*_*mod*_ = (1 + *C* − 0.5 · *O* − *S* − 0.5 · (*N* + *P* + *H*))/(*C* − 0.5 · *O* − *S* − *N* − *P*)) and the nominal oxidation state of carbon (NOSC = 4 − [(4C + H − 3 N − 2O − 2S)/C])^40,55,56^. All molecular formulae were further associated with different previously described molecular groups (e.g., peptide- or polyphenol-like, highly unsaturated O_rich_/O_poor_, unsaturated aliphatics, saturated fatty acids and sugars)^57^. The number of formulas in the dataset was then further reduced with a dedicated R script. First, formulas detected in the original solvents of the analyzed mixture (methanol and ultrapure water) were deleted, but only if the associated intensity was lower than in the highly diluted samples. All formulas were removed that did not meet the following restrictions: C ≥ O; O > (2P + S); H ≤ 2C + 2; N ≤ 6; S ≤ 2 and P ≤ 1. Assuming a close to gaussian distribution, masses were removed if their intensity exceeded by a factor of more than 2 from the maximum intensity among the preceding and succeeding 10 formulae in the mass spectra (spectral smoothing). The remaining list was further reduced by removing several known contaminants. Next, only those formulas that were detected in both of the replicated measurements were to remain in the dataset with an averaged intensity assigned. Finally, only formulas were considered that occurred in at least 2 different samples. Visualization and further evaluation of the SPE-DOM data was based on two versions of the dataset. One that contained all remaining formulas and another, in which each sample was limited to the individual 1000 formulae with the highest signal intensities. The dataset with all formulae was used to calculate intensity weight averages of the aforementioned molecular categories, indices, ratios and elemental counts for each sample following a normalization to total intensity (see Supplementary Table S1). The degradation index I_DEG_ as well as functional diversity indices D_F_(C), D_F_(H/C) and D_F_(NOSC) were also calculated based on this dataset^37,42^. I_DEG_ was calculated according to Flerus et al.^37^ by relating the raw intensities of specifically identified index formulae that are negatively (C_21_H_26_O_11_, C_17_H_20_O_9_, C_19_H_22_O_10_, C_20_H_22_O_10_, C_20_H_24_O_11_) or positively (C_13_H_18_O_7_, C_14_H_20_O_7_, C_15_H_22_O_7_, C_15_H_22_O_8_, C_16_H_24_O_8_) correlated with radiocarbon age (I_DEG_ = Σ (magnitudes NEG_IDEG_)/Σ (magnitude (NEG_IDEG_ + POS_IDEG_))^36^. Functional diversity indices for each sample were derived by relating individual intensity products (*p*) of consecutive formulae (*i*, *j;* with regards to mass) to differences in a respective functional parameter (*c* = C | H/C | NOSC) following a previously published equation (Eq. 1; implemented as an R script)^41^.

1 $${D}_{F}\left(c\right)= \sum_{i=1}^{N-1}\sum_{j=i+1}^{N}{p}_{i}\bullet {p}_{j}\bullet \left|{c}_{i}-{c}_{j}\right|$$

These averaged values were then compared to other parameters including [Fe], ligands, DOC, TDN and microbial community systematics. Statistical analyses were conducted, including calculation of Bray–Curtis dissimilarities (Vegan package, R)^58^ between the samples and used for a Principal Coordinate Analysis (PCoA) to visualize the variability of the data. The dataset limited to 1000 formulae per sample was used to find further Pearson and Spearman Rank correlations between SPE-DOM characteristics and microbial community and other environmental data. Derived SPE-TDN concentrations in the samples from incubations 0.1 µM Fe III, 10 µM Fe I and 100 µM Fe I were higher than total TDN. As this is a clear sign for contamination of the SPE-DOM extract, the respective samples were excluded from the following DOM characterization.

### DNA and RNA extraction, cDNA generation, amplification of 16S tags, and sequencing

DNA and RNA extraction, cDNA generation, amplification of 16S tags, and sequencing were performed as has been described before (Böhnke et al.^60^). Briefly, DNA was extracted from half of the filter of the 053 CTD 04 sample using the DNeasy PowerSoil Kit, (Quiagen, Venlo, Netherlands) as specified by the manufacturer (24 ng µL^−1^ with a 260/280 ratio of 1.44). RNA was extracted from half of a polycarbonate filter of all previously mentioned ligand experiment samples using the Direct-zol™ RNA Miniprep Plus Kit (Zymo, Irvine, CA, USA) following the manufacturer’s instructions. Total DNA removal was ensured by performing a second DNaseI digestion step using the DNase Max kit (Quiagen), according to the provided protocol (1.1 and 1308.7 ng µL^−1^). Up to 2.5 µg of the isolated RNA was used as starting material for the synthesis of cDNA with Invitrogen’s SuperScript® VILO™ cDNA Synthesis Kit (Life Technologies™, Darmstadt, Germany), according to the manufacturer's protocol (11.1 and 472.8 ng µL^−1^ with a 260/280 nm ratio ranging from 1.71 to 1.94).

Generated cDNA served as a template (5 ng) for paired-end 16S rRNA gene sequencing on an Illumina MiSeq platform like it has been described before^59,60^. In short, the (i) hypervariable V3 and V4 regions of the bacterial and the (ii) hypervariable V4 and V5 regions of the archaeal 16S rRNA gene were amplified using the following primer pairs: (i) S-D-Bac-0341-b-S-17 and S-D-Bac-0785-a-A-21 for bacterial 16S tags^61^ and (ii) Arch_519F and Arch 915R^62^, as well as Arch 524F and Arch 958R^63^ for archaeal 16S tags. Two parallel PCR reactions were performed for each of the three primer sets (in total 2 × 3 PCRs per sample) using the Kapa Hifi HotStart Ready Mix (Kapa Biosystems, Boston, MA, USA) according to manufacturer’s instructions. PCR conditions were set as follows: 3 min initial denaturation followed by 25 cycles of denaturation at 95 °C for 30 s, annealing at 55 °C and 63 °C for amplification of the bacterial and the archaeal 16S rRNA gene, respectively, and extension at 72 °C for 30 s. After pooling the 1 × 2 parallel samples of the bacterial 16 s rRNA gene amplification and the 2 × 2 parallel samples of the archaeal 16S rRNA amplification purification was done using the Agencourt AMPure XP beads (Beckman Coulter, Brea, CA, USA) according to manufacturer’s protocol. In a subsequent amplification step multiplexing indices and Illumina® sequencing adapters were added by using the Nextera® XT Index Kit (Illumina®, San Diego, CA, USA) and the Kapa Hifi HotStart Ready Mix (Kapa Biosystems) under the same PCR conditions mentioned above but with 8 cycles. After the indexed samples were purified (Agencourt AMPure XP beads, Beckman Coulter) they were pooled in a way that all subsamples contained an equimolar amount of 16S rRNA gene amplificate. This library pool was then analyzed by the 2100 Bioanalyzer (Agilent Technologies, using the DNA High Sensitivity Chip). The amplicon libraries were sequenced by paired-end sequencing in a 2 × 300 bp run on the MiSeq platform (Illumina, St. Diego, USA).

### Analyses of 16S tags

Sequences were processed using the Qiime2 environment^64^. The filtering and merging of demultiplexed raw reads were performed using the dada2-plugin with default settings and removal of the primer sequences^65^. Samples with less than 1000 sequences after merging were not further analyzed (except for the environmental sample, not subjected to incubation experiments). For taxonomic assignments the SILVA database release 138^66^ was pretrained with the respective primer pairs for Bacteria and Archaea^67,68^. The assignments were computed using the feature-classifier plugin (classify sklearn) with default settings and the pre-trained SILVA classifier^68^. The phylogeny was calculated using the “align-to-tree-mafft-fasttree” pipeline and rarefaction curves were calculated with the qiime diversity alpha-rarefaction visualizer using a sampling depth of 10,300 sequences^69,70^. Principle Coordinate Analysis (PCoA) was performed using the core-metrics-phylogenetic pipeline with a sampling depth of 1500^71,72^.

## Supplementary Information

Supplementary Information 1.

Supplementary Information 2.

Supplementary Information 3.

Supplementary Information 4.

## Data Availability

Sequence data was deposited at the SRA (Sequence Read Archive) of NCBI (National Center for Biotechnology Information) under the BioProject 689,485.
