# Supplementary material for: Impact of high Fe-concentrations on microbial community structure and dissolved organics in hydrothermal plumes: an experimental study
Source: Sci Rep. 2022 Dec 1;12:20723. doi: 10.1038/s41598-022-25320-0 (PMC9715565; doi:10.1038/s41598-022-25320-0)
Supplement: Supplementary file 1 — Supplementary Information 1. [file 41598_2022_25320_MOESM1_ESM.docx]

***Supplementary Information for:***

**Impact of high Fe-concentrations on microbial community structure and dissolved organics in hydrothermal plumes – an experimental study**

Christian T. Hansen^1,2*^, Charlotte Kleint^2,3^, Stefanie Böhnke^4,5^, Lukas Klose^3,2^, Nicole Adam-Beyer^4,5^, Katharina Sass^5^, Rebecca Zitoun^7,8^, Sylvia G. Sander^8^, Daniela Indenbirken^6^, Thorsten Dittmar^1,2^, Andrea Koschinsky^2,3^ and Mirjam Perner^4,5^

^1^Institute for Chemistry and Biology of the Marine Environment (ICBM), Carl von Ossietzky University of Oldenburg, Germany

^2^Center for Marine Environmental Sciences (MARUM), University of Bremen, Germany

^3^Department of Physics & Earth Sciences, Jacobs University Bremen, Germany

^4^Geomicrobiology, Department of Marine Biogeochemistry, GEOMAR Helmholtz Centre for Ocean Research, Kiel, Germany

^5^previously: Molecular Biology of Microbial Consortia, Biocenter Klein Flottbek University of Hamburg, Germany

^6^Heinrich-Pette-Institut, Leibniz Institute for Experimental Virology, Martinistraße 52, 20251 Hamburg, Germany

^7^ Department of Chemistry, University of Otago, Dunedin 9054, New Zealand

^8^ Marine Mineral Resource Group, GEOMAR Helmholtz Centre for Ocean Research Kiel, 24148, Kiel, Germany

*Corresponding Author: Christian Hansen, Center for Marine Environmental Sciences (MARUM), University of Bremen, Klagenfurter Straße 2-4, 28359 Bremen, Germany, Phone: (49) 421 218 65405, [chansen@marum.de](mailto:chansen@marum.de)

**This file contains:**

Supplementary Results S1 – S2

Supplementary Table S1 – S5

Supplementary Figure S1 - Figure S2

*Supplementary Results:*

**Supplementary Results S1**

**Implications in context of ligand stabilities.** Conditional stability constants (logK_i_^cond^) were determined for the Fe-binding organic ligands but were not used in the manuscript’s main discussion. However, they are included here as additional information that might be of interest. Bond strength of Fe-binding complexes appear to be anticorrelated with the ligand to dFe ratio (see Table 1). Potentially other ligands with lower logK values are produced in higher dFe incubations, which could reflect the observed deviating microbial community at these conditions. If we assume that microbes produce ligands in order to prevent cell encrustation at potentially toxic levels of dFe, it can be speculated that up to an dFe concentration of 1 µM, the microbes managed to produce an increasingly larger excess of ligands to cope with increasing levels of dFe. At higher dFe levels, however, ligand production efficiency decreases, consistent with the marked shift in the microbial community composition, which now appears to be made up of microbes with altered ligand-forming properties.

**Supplementary Results S2**

**Variations in archaeal community structure along an Fe gradient.** Very few archaeal sequences could be obtained from the microcosm incubations (Supplementary Figure S2) and may indicate that nutrients important for archaeal growth were not sufficiently present. For 8 samples, however, enough sequence reads were found even after the cut off was set at 1000. These were incubation samples 0 µM III, 0.1 µM I and III, 1 µM I and II, 10 µM III, 100 µM III, and the plume sample. *Nitrosopumilaceae* were the prevailing Archaea in the naturally occurring plume fluid (98%) and in all evaluable incubations (91-100%). Marine Group III *Thermoplasmata* appear in small amounts in three incubations (0.1 µM I, 1 µM II and 10 µM, 5.3%, 1.3% and 0.3%, respectively) but is absent in the original plume sample and in the 0 µM Fe spiked incubation likely indicating that their growths is supported by low Fe contents. Same holds true for the Marine Benthic Group A which is also absent in the plume sample and in the 0 µM Fe spiked incubation but in low numbers present in 2 incubations (1 µM I and 100 µM III, 2.6% and 1.4%). Marine Group II made up of 1.3% of the archaeal community in the original plume sample and is absent in all incubations indicating that *in vivo* conditions are not supportive for their growth. At higher Fe levels of 1 mM and 10 mM no archaeal sequences were found likely reflecting the toxicity of high Fe concentrations for local archaeal representatives and their inability of dealing with high Fe contents.

*Supplementary Tables:*

**Supplementary Table S1.** Reduced dataset (see †, ‡, • and * in Table 1) for SPE-DOM (contaminated and inconclusive samples removed, see methods for details).

**Supplementary Table S2.** Fe containing organic formulae and average characteristics for reduced dataset (see †, ‡, • and * in Table 1). Only formulae that occurred in all replicates of each incubation set (section).

**Supplementary Table S3.** Cross table with all formulae for which relative intensity correlates positively and negatively with relative abundance of different microbial groups (Spearman rank correlation; p < 0.05).

**Supplementary Table S4.1.** Fe containing formulae that correlate positively / negatively with certain microbial groups and dFe concentration (Pearson correlation; p < 0.05).

**Supplementary Table S4.2.** Cross table with all Fe-formulae for which relative intensity correlates positively and negatively with relative abundance of different microbial groups (Pearson correlation; p < 0.05).

**Supplementary Table S5.** Ligand and conditional stability constants, logK_i_^cond^ with errors.

| **Sample** | **dFe (µM)** | **L (µM)** | **+/- L** | **error %** | **Log K** | **+/- log K** |
| --- | --- | --- | --- | --- | --- | --- |
| **Plume sample (P)** | 0.071 | 0.214 | 0.104 | 49.0 | 18.86 | 0.280 |
| **Control (C) II** | 0.295 | 0.324 | 0.002 | 0.600 | 22.15 | 0.055 |
| **Inc 0 µM Fe I** | 0.225 | 0.421 | 0.051 | 12.0 | 20.58 | 0.154 |
| **Inc 0.1 µM Fe I** | 0.201 | 0.431 | 0.037 | 9.0 | 20.00 | 0.080 |
| **Inc 1 µM Fe I** | 0.964 | 3.09 | 0.261 | 8.0 | 19.50 | 0.060 |
| **Inc 10 µM Fe III** | 10.70 | 27.0 | 2.29 | 8.0 | 20.20 | 0.070 |
| **Inc 100 µM Fe II** | 107 | 239 | 60.0 | 25.0 | 19.50 | 0.170 |
| **Inc 1 mM Fe II (‡)** | 1000 | 1291 | 74.7 | 6.0 | 21.26 | 0.130 |

*Supplementary Figures:*

**Supplementary Figure S1**


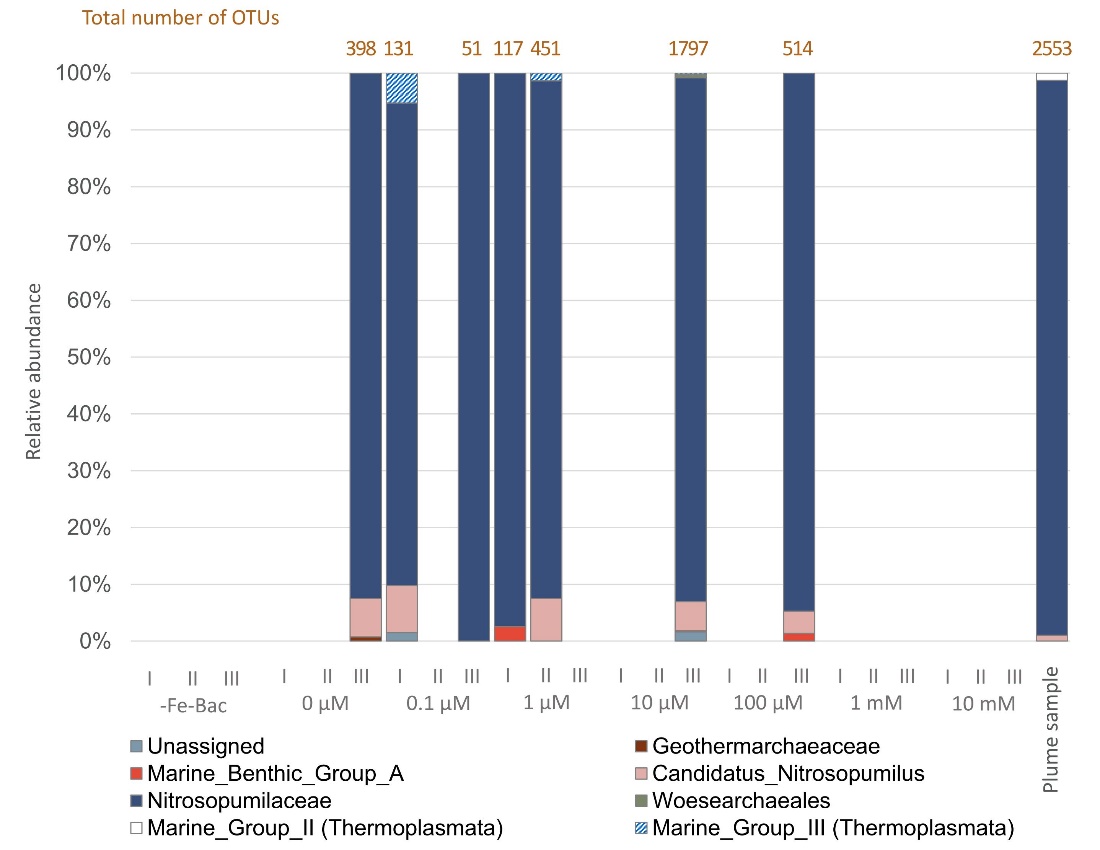


Taxonomy plots showing relative abundances of archaeal 16S rRNA tags from plume incubation experiments along an Fe gradient. No cut-off for total number of OTUs were set. Samples without taxonomy plots thus had zero sequence reads.

**Supplementary Figure S2**

**
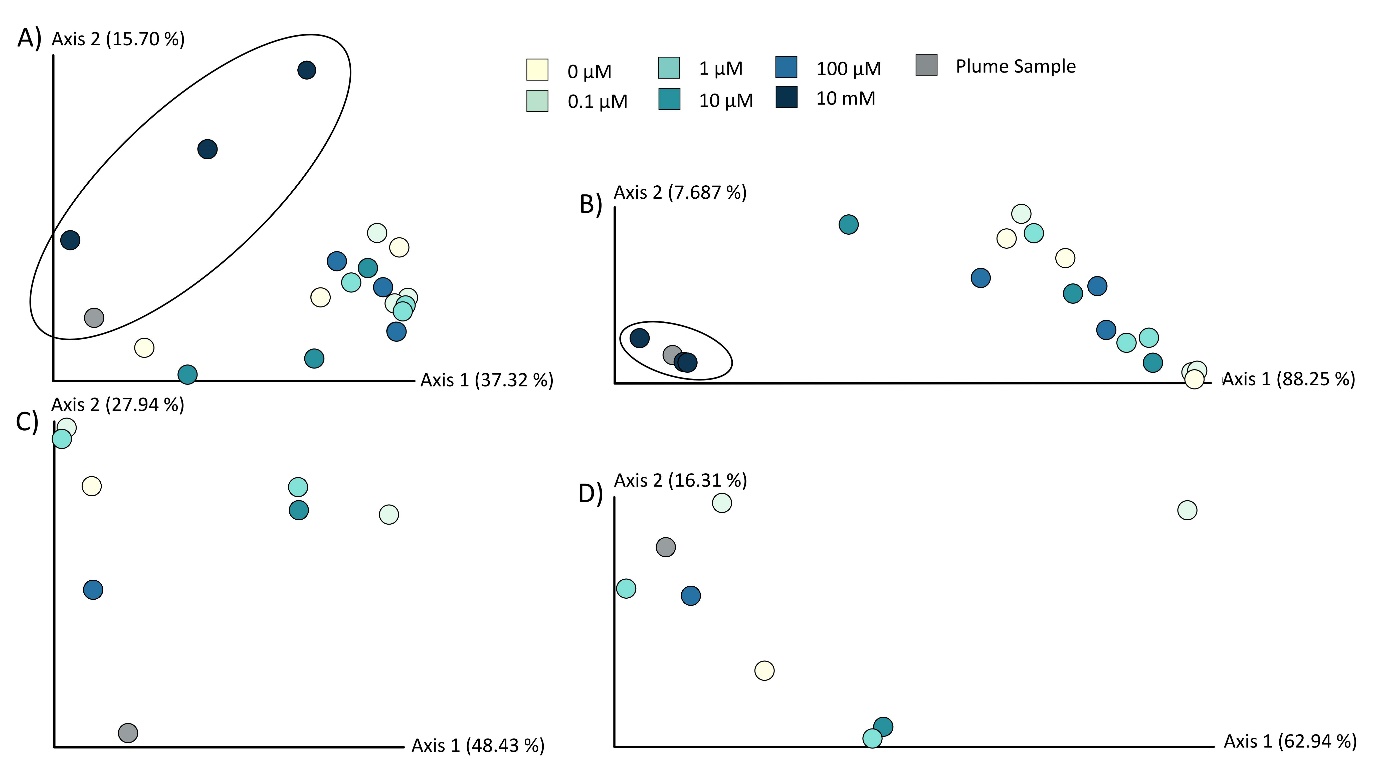
**

PCoA analysis of bacterial (A, B) and archaeal (C, D) communities of incubations along a Fe-gradient using the weighted (A, C) and unweighted (B, D) UniFrac distance metric. Sequences were processed using the Qiime2 environment (Bolyen et al., 2019). Filtering and merging were performed using the dada2-plugin with default settings, the phylogeny was calculated using the “align-to-tree-maffft-fasttree pipeline and PCoA was performed using the core-metrics-phylogenetic pipeline with a sampling depth of 10300
